# Thank you to *The Lancet Regional Health - Southeast Asia’s* clinical and statistical peer reviewers in 2023

**DOI:** 10.1016/j.lansea.2024.100367

**Published:** 2024-02-09

Jaffar Abbas

Mohammad Abdollahi

Safura Abdool Karim

Zainab Abdulrasol

Rajib Acharya

Monica Violeta Achim

Bipin Adhikari

Biplov Adhikari

Tulsi Adhikari

Adewale Adisa

Brian Adkins

Joel Adler

Girdhar Gopal Agarwal

Sandip Agarwal

Preeti Aggarwal

Azeem Ahmad

Khabir Ahmad

Bilal Ahmed

Farooq Ahmed

Imtiaz Ahmed

Rashida Ahmed

Tanvir Ahmed

Tausif Ahmed

Gulzhanat Aimagambetova

Dewi Aisyah

Elizabeth Helen Aitken

H Ajay Kumar

Sheikh Mohammad Fazle Akbar

Pooya Alaedini

K Alagarasu

Iqra Alamgir

Mariam Al-Ashbal

Barnabas Alayande

Natasha Ali

Syed Mustafa Ali

Fatima Almaghrabi

Arshad Altaf

Gabriela Gonçalves Amaral

Ayush Anand

Blake Angell

Dr Abhishek Anil

Danae Apatzidou

S M Yasir Arafat

Libnah Areias

Aashima Arora

Grace Arteaga

Thirunavukkarasu Arun Babu

Muhammad Aslam

Anushka Ataullahjan

Aditya Athotra

Alok Atreya

Htin Lin Aung

Modupe Alake Ayoade

Karan Babbar

Bontha V Babu

Priya Baby

Bhavani Shankara Bagepally

Shasha Bai

Kaushalya Bajpayee

Muthuswamy Balasubramanyam

Ramesh Banadakoppa Manjappa

Dipanwita Banerjee

Susovan Banerjee

Avi Bansal

Sukhadeo B Barbuddhe

Mary Barker

Kanksha Barman

Alfred Bartolucci

Pranjali Bawankar

Shahina Begum

Deepak Behera

Reinout Bem

James Berkley

Nandita Bhan

Aneel Bhangu

Mausumi Bharadwaj

Omesh Bharti

Javeed Bhat

Gayatri Bhatia

Shinjini Bhatnagar

Atanu Bhattacharjee

Shampa Bhattacharjee

Vishal Bhavsar

Sandesh Bhusal

Zulfiqar Bhutta

Gabriel Birgand

Praveen Birur N

Isha Biswas

Jheelam Biswas

Aaron W. Bivins

Micheal Boachie

Matteo Boattini

Gerry Bodeker

Melanie Boeckmann

Kaushik Bose

Mampi Bose

Farid Boumédiène

Thorsten Braun

Carl Britto

Eva Brun

Nilesh Buddha

Atul Madhukar Budukh

Warwick Butt

Ewan Cameron

Cheryl Carcel

Rodrigo Cataldi

Sankha S Chakrabarti

Suman Chakrabarti

Suman Chakrabarty

Maisuri Chalid

Karim Chamari

Ko Ling Chan

Yiong Huak Chan

Rahul Chanchlani

Arun Chandrasekharan

Pankaj Chaturvedi

Vijay Kumar Chava

I Ming Chen

Jinjie Chen

Ying-Yeh Chen

Li Cheung

jagadish Chhetri

Yook-Chin Chia

Ka Chun Chong

Rijo Mathew Choorakuttil

Supriya Chopra

Abhijit Chowdhury

Md Nabidul Haque Chowdhury

Cindy Chu

Aurauma Chutinet

Karly Cini

Kajal T Claypool

Tammy Clifford

Andreu M Climent

Abu Conteh

Andrew Copas

David Cromwell

Caroline Daccache

Minakshi Dahal

Victor Dahlblom

Rakhi Dandona

Masoud Dara

Arijit Das

Bhudev Das

Kausik Das

Samir K Das

Sayan Das

Sushmita Das

Rajib Dasgupta

Kapilkumar Dave

Julie Davies

Erol Demir

Heena Desai

Anupam Dey

Ravindran Dhanapal

Sivashanmugam Dhandapani

Puneet Dhar

James Diffey

Saber Dini

Gauri Divan

Matthieu Domenech de Cellès

Amol R Dongre

Sithar Dorjee

Wellington dos Santos

Britt I Drögemöller

Yanqiu Du

Aditya Kumar Dubey

Tanja Dujić

Karthickeyan Duraisamy

Kevin Durr

Amit Dutta

Sourabh Dutta

Gaurav Raj Dwivedi

James A Ellison

Omer Elneima

Talha Bin Emran

María Fernanda Escobar-Vidarte

Paolo Eusebi

Joseph Fadare

Mochammad Fahlevi

Asima Faisal

Boye Fang

Hossein Farrokhpour

Forough Farrokhyar

Muhammad Junaid Farrukh

Syeda Sadia Fatima

Bruno Fattizzo

Shaffi Fazaludeen Koya

Dolorosa Fernandes

Aitziber Fernández-Jiménez

David Flood

Anjali Forber-Pratt

Anna Freni Sterrantino

Han Fu

Sunyang Fu

Ryoma Fukuoka

Anita Gadgil

Sailaxmi Gandhi

Mihir Gandhi

Trivadi Ganesan

Aparna Gangopadhyay

Nirmal Ganguly

Nuno Garcia

Suneela Garg

Tushar Garg

Dominic Gasbarro

Sneha Gautam

Ezra Gayawan

Georges Gebrael

S Ghatak

Ravindra Bhaskar Ghooi

Dhruva Ghosh

Nilanjana Ghosh

Uday Ghoshal

Michael Gibson

Mary Ellen Gilder

Justin Gill

Emmanuelle Girodon

Camila Giugliani

Melissa Gladstone

Ronald S Go

Akshay Goel

Gyanendra N Gongal

Ram Gopalakrishnan

Shivkumar Gopalakrishnan

Radhika Gore

Vishal Goyal

Cai Grau

John Gregson

Anneke Grobler

Nachiket Gudi

Kamal Gulati

Alistair Gunn

Harish Gupta

Rishab Gupta

Subhash Gupta

Yashdeep Gupta

Mohan Gupte

Kapil Gururangan

Faisal Hakeem

Masahide Hamaguchi

Shaffa Hameed

Raph Hamers

Muhammad Haroon Hamid

Budi Haryanto

Ahmar H Hashmi

Divna M Haslam

Md Sharif Hassan

Karin Haustermans

Yan He

Yulei He

Manupati Hemalatha

David Heymann

Aniqa Tasnim Hossain

Mohammad Sorowar Hossain

Muhammad Anwar Hossain

John Howard

Natasha Howard

Mila Nu Nu Htay

Ming Huang

Yute Huang

Kevin K C Hung

Sartaj Hussain

Tahziba Hussain

Kumaravel Ilangovan

Ana-Maria Iosif

Farah Ishtiaq

Meltem Işıkgöz Taşbakan

Md Islam

Towfiqul Islam

Priya Iyer

Bart Jacobs

Rahul Jahagirdar

AmitaJain Jain

Naveen Jain

Md Jakariya

Rajiv Janardhanan

Kana Ram Jat

Pabitra Jena

Joshua Jeong

Amar Jesani

Vivekanand Jha

Pranav Joshi

Udita Joshi

Lakshmi Josyula

Desmond Jumbam

Seyram Kaali

Abdulla Al Kafy

Anuska Kalita

Sureshkumar Kamalakannan

Mohammad Karamouzian

Abhilasha Karkey

Subhradip Karmakar

Mohmed Isaqali Karobari

Shilpa Karvande

Geetanjali Mahagundappa Katageri

Shivanand Kattimani

Anand Kawade

Masataka Kawamura

Pearse Keane

Mark Kelson

Zoe Kelson

Samantha Keogh

Marko Kerac

Sushmita Kerketta

Vikash Keshri

Kadircan Keskinbora

Anuradha Khadilkar

Amjad Khan

Ashraful Islam Khan

Ejaz Ahmad Khan

Maroof Khan

Md. Abdullah Saeed Khan

Md. Nuruzzman Khan

Muhammad Khan

Shakir Khan

Sudhir Khanal

Gulam Mustofa Khandaker

Rajeshwar Khandare

Sukhanshi Khandpur

Vasudha Khanna

Chin Meng Khoo

Jung-wook Kim

Uday Kiran

David K Klassen

Andrea König

Evangelos Kontopantelis

Reynaldi Ikhsan Kosasih

Michał Kościółek

Praew Kotruchin

Rajkumar Kottayasamy Seenivasagam

Yuvaraj Krishnamoorthy

Evangelos Kritsotakis

Frédéric Krumbein

Omar Kujan

Rajesh Kulkarni

Anil Kumar

Jogender Kumar

Kaushal Kumar

Kishore Kumar

Paninjukunnath Ravi Kumar

Rahul Kumar

Renuka Kunte

Nirmal Kurian

Yohannes Kurniawan

Keisuke Kuroda

Vivek Kute

Vellappillil Kutty

Hye-Young Kwon

Myat Phone Kyaw

Thomas Lacoste-Palasset

Chandrakant Lahariya

Preet Lal

Sze Tung Lam

Tella Lantta

Justin T Lawson

Lisa Lazarus

Philip Lazarus

Yong-ho Lee

Hemkhothang Lhungdim

Li Li

Mengmeng Li

Philip Li

Jiawen Liao

Raquel Lima

Julian Little

Qi Liu

Xiaoning Liu

Xin Liu

Peter Lloyd-Sherlock

Kavita Lole

Reza Lotfi

Guangyu Lu

Mirjam Lukasse

Alain Luxembourg

Barbara Madaj

Pavithra S Madamarandawala

Dhammika Magana Arachchi

Mehrsadat Mahdizadeh

Tathagata Mahintamani

Nasir Mahmood

Sharada Mailankody

Govind Makharia

Asrar Malik

Manal Malik

Hassen Mamo

Mohammed Mamun

S Manikandan

Prabhu Manivannan

F Marcon Alfieri

Russell Seth Martins

Melvin Marzan

Bruno Masquelier

Shahril Azian Haji Masrom

Francesco Massari

Sri Masyeni

Céu Mateus

Roger Mathisen

Prashant Mathur

Tomohiro Matsuda

Fiona Matthews

Nekma Meah

Gajendra Kumar Medhi

Jagdish Meena

Man Mohan Mehndiratta

Ravi Mehrotra

Abhishek Mehta

Ritu Mehta

Hagazi Gebre Meles

Antonella Meloni

Martin I Meltzer

Satish Mendonca

Fiona Mensah

Michela Meregaglia

Stefan Michiels

Melissa Middleton

Katja Mielke

Huynhvan Minh

Masashi Misawa

Arnab Kumar Mishra

Rakesh K Mishra

Dipak Mitra

Prasenjit Mitra

Umar Muhammad Modibbo

Shooka Mohammadi

Viswanathan Mohan

Itismita Mohanty

Pratap Mohanty

Dinabandhu Mondal

Nachiket Mor

Ghobad Moradi

Michelle Morales

Greg Moran

Michael L Moritz

Taslin Jahan Mou

Satish Mudiganti

Debjani Mueller

Abdul Razzaq Mughal

Diptadhi Mukherjee

Arnab Mukherji

Ivan Müller

V R Muraleedharan

Jill Murphy

Zia Ul Mustafa

Rohitha Muthugala

Asaad Nafees

Geeta Sowmya Narayanan

Gustavo G Nascimento

Naser Nasiri

Martina W S Nasrun

Salwa Naushin

Janardhana Navaneetham

Sadiq Naveed

Hasan Nawaz

Rashad Nawfal

Prakash Negi

Dinesh Neupane

Joshua Ng-Kamstra

Hieu Ngo

Amanda Nguyen

Tu Nguyen

Maria Carmen Nievera

Kayzad Nilgiriwala

P Lakshmi Nirisha

Chayanin Nitiwarangkul

John Norrie

Bernard North

Kate Northstone

Jean Jacques Noubiap

Samuel Nsobya

Gilbert Sterling Octavius

Daniel Oesterle

Charity Oga-Omenka

Tatsuya Ohno

Shalini Ojha

Omotayo Olaoye

Tom W Ouellette

Isao Oze

Ramachandran Padmavati

Madhukar Pai

Pankaj Kumar Panda

Rajmohan Panda

Achyut Raj Pandey

Manoj Pandey

Neeraj Pandey

Apurva Kumar Pandya

Vikas Panibatla

Krishna Pant

Anupam Parashar

Arpit Parmar

Shahila Parween

Patrizio Pasqualetti

Daniele Pastori

Prakash Patel

Ranjan Kumar Patel

Shivani Patel

Vijay Patil

Paul Patinadan

Somdatta Patra

Chitra Pattabiraman

Jay R Patwa

Elise Pauzé

Nicola Pavan

Dharshani Pearson

Petrick Ramesh A L K Periyasamy

Dyah Aryani Perwitasari

Michael Peters

Huong Pham

Sirinya Phulkerd

L Pistoia

Yashashwi Pokharel

Marko E Popović

Tushar Prabhakar

Kumar Prabhash

Sarbeswar Praharaj

Shantanu Prakash

Gadde Praveen

Anuja Premawardhena

Robin Prescott

Rakesh PS

Samuel J Pullen

Meera Purushottam

Ejaz Qadeer

Soghra Rabizadeh

Venkatraman Radhakrishnan

Kazi Rahman

Md Siddikur Rahman

T Rajapakse

Eileen Rakovitch

Astha Ramaiya

Lakshmy Ramakrishnan

Sudha Ramani

Harish Ranjani

Jonas Ranstam

Annemarei Ranta

Girish Rao

Neha Rastogi

Chaturbhuj Rathore

Bevinahalli Nanjegowda Raveesh

Manickam Ravichandran

Ajaree Rayanakorn

Muhammad Rehman

Gouroumourty Revadi

Henry Rice

Tyler hyungtaek Rim

Bayard Roberts

Andrea Rodriguez

Minakshi Rohilla

Britta Rosenberg

Reshma Roshania

Jeremy Ross

Geoffrey Rossi

Wolfgang A Rottbauer

Arpita Nibedita Rout

Shannon Ruzycki

Gayatri Saberwal

Banshi Saboo

Harshpal Sachdev

Rajeev Sadanandan

Balakrishnan Sadasivam

Sahar Saeedi Moghaddam

Marc Saez

Ranjit Sah

Avijit Saha

Tushar Sahasrabudhe

Krushna Sahoo

Swapnajeet Sahoo

Kirti Sundar Sahu

Saroj Sahu

Sanjiv Saigal

Hidehisa Saito

Sheikh Saleem

Zikria Saleem

Lubna Samad

Elena Samonova

Laura Sampietro-Colom

Rengaswamy Sankaranarayanan

Hirozumi Sano

Pairoj Saonuam

Vishnu Prasad Sapkota

Fred Sarfo

Swarup Sarkar

Shafiqul Alam Sarker

Rakesh Sarwal

R Tedjo Sasmono

Mohammed Abdus Sattar

Naveed Sattar

Jitendra Pal Singh Sawhney

Vartika Saxena

Elizabeth Schieber

Jonathon P Schubert

David T Selewski

Kalaiselvi Selvaraj

Tarun K Sen Gupta

Manju Sengar

Graham Serjeant

Shekhar P Seshadri

F A Shafie

Daksha Shah

Komal Shah

Sharaf Ali Shah

Bhaskar Shahbabu

Sadia Shakoor

Mohammadreza Shalbafan

Rajesh Shankar Iyer

Mike Sharland

Deepak Sharma

Mithun Sharma

Nandini K Sharma

Pawan Sharma

Shailja Sharma

Swati Sharma

Digant D Shastri

Jayanthi Shastri

Donna R Shelley

Zumin Shi

Takuya Shiga

Sunil Shrestha

Zubin Cyrus Shroff

Hidayet Sıddıkoğlu

Kamran Siddiqi

Sameen Siddiqi

Saulat Siddique

Pooja Sikka

Santhi Silambanan

Gisele Silva

Bryony Simmons

Joana F F Simões

Rodney Sinclair

Abhishek Singh

Gagandeep Singh

Gagandeep Singh

Nishikant Singh

Ruchi Singh

Shalini Singh

Swarndeep Singh

Monika Singla

Avik Sinha

Baerbel Sinha

Bhawna Sirohi

Todd G Smith

A Somasundaram

Kapil Dev Soni

Arjun Srinivasan

Shobhit Srivastava

Prakash SS

Ruby Stocker

Alice Street

Ramnath Subbaraman

Deepak Subedi

Karthick Subramanian

Alka Anand Subramanyam

Ratna M Sudarshan

Nikkil Sudharsanan

Melissa Sutton

TarunKumar Suvvari

Sabyasachi Swain

Imdadul Talukdar

Jackson Tan

Kok Hian Tan

Mihoko Tanabe

Ajay Tandon

Rohit Tandon

Julian Tang

Marjan Tariverdi

Lambed Tatah

Walter Taylor

Hadi Tehrani

Berna Terzioğlu Bebitoğlu

Jeromie Wesley Vivian Thangaraj

Rangaswamy Thara

Harish Thippeswamy

Usharani Thirunavukkarasu

Susan Thomas

Vivien Thomson

Kirkby Tickell

Shiyam Sunder Tikmani

Klara Tisocki

Stephanie Topp

Hidenori Toyoda

Phyllida Travis

Srikanth Tripathy

Wai Chung Tse

Pham Tuan

Hein Tun

Hannele E Turunen

Bhishma Tyagi

Veronica Ueckermann

Snekhalatha Umapathy

N A Uvais

Viorela Ligia Vǎidean

Pradeep Vaideeswar

Girija Vaidyanathan

Janhavi Ajit Vaingankar

Camilo E Valderrama

David van de Vijver

Hanneke Van Laarhoven

Abi Tamim Vanak

Kristin E VanderEnde

Jithin Varghese

Priya Vart

Rajani Ved

Andres Velasco-Villa

Padmasani Venkatramanan

Baskar Venkidasamy

Francesco Venturelli

Shweta Jain Verma

Tanuwong Viarasilpa

Lakshmi Vijayakumar

Patricia Villain

Bella Vivat

Matthew John Wade

Abdul Waheed

Josephine Walker

Judd Walson

Eric Yuk Fai Wan

Carol A Wang

Yihang Wang

Youhua Wang

Xi Wei

Paul Weiss

Mengtzu Weng

Matthew D Westercamp

Wit Wichaidit

Indah Widyahening

Phoebe C M Williams

Georg Wolff

Yiu Ming Wong

Kwankaew Wongchareon

Limin Xing

Lei Xu

John Yabe

Anita Yadav

Arun Yadav

Chittaranjan Yajnik

Nadir Yalçın

Gowri Yale

Hiroyuki Yamada

Weihui Yan

Muhammad Yani

Sandul Yasobant

Sandhya Kanaka Yatirajula

Eng-Kiong Yeoh

Seda Yerlikaya

Mehmet Yilmaz

Taishi Yonetsu

Saengduean Yotanyamaneewong

Mohammad Tahir Yousafzai

Seyede Raheleh Yousefi

Audrey Yue

Cindra Tri Yuniar

Evy Yunihastuti

Siddhesh Zadey

Ashkan Zandi

Shah Zeb Khan

Hajo Zeeb

Ayse Zengin

Fengqing Zhang

Xun Zhuang

